# Applying synchrotron radiation-based attenuated total reflection-fourier transform infrared to chemically characterise organic functional groups in terrestrial soils of King George Island, Antarctica

**DOI:** 10.1016/j.heliyon.2023.e19711

**Published:** 2023-08-31

**Authors:** Siwatt Pongpiachan, Kanjana Thumanu, Chulalak Chantharakhon, Chunmanus Phoomalee, Teetat Charoenkalunyuta, Kittiphop Promdee, Saran Poshyachinda, Muhammad Zaffar Hashmi

**Affiliations:** a NIDA Center for Research & Development of Disaster Prevention & Management, School of Social and Environmental Development, National Institute of Development Administration (NIDA), 148, Sereethai Road, Klong-Chan, Bangkapi, Bangkok, 10240, Thailand; b Synchrotron Light Research Institute (Public Organisation), 111 Moo 6, University Avenue, Muang District, Nakhon Ratchasima, 30000, Thailand; c Department of Survey Engineering, Chulalongkorn University, Bangkok, 10330, Thailand; d Department of Environmental Science, Chulachomklao Royal Military Academy, Nakhon Nayok, 26001, Thailand; e National Astronomical Research Institute of Thailand (Public Organization) 260 Moo 4, T. Donkaew, A. Maerim, Chiang-Mai, 50180, Thailand; f Department of Chemistry, COMSATS University Islamabad, Islamabad, Pakistan

**Keywords:** SR-ATR-FTIR, Terrestrial soils, Organic functional groups, King George Island, Chemical pollution

## Abstract

Anthropogenic activities, especially associated with fossil fuel combustion, are raising concerns worldwide, but remote areas with extreme climate conditions, such as Antarctica, are isolated from the adverse influence of human civilisation. Antarctica is considered as the most untouched place on Earth. Such pristine areas, which have extremely low chemical pollutant concentrations owing to restricted anthropogenic impacts, exemplify plausible model environments to test the reliability and sensitivity of advanced analytical techniques employed to chemically characterise and evaluate the spatial distribution of chemical pollutants. Here, synchrotron radiation-based attenuated total reflection-Fourier transform infrared (SR-ATR-FTIR) spectroscopy was employed to evaluate the variations in the organic functional groups (OFGs) of terrestrial soils of King George Island, Antarctica. Second-derivative SR-ATR-FTIR spectroscopy coupled with several multivariate statistical techniques highlighted the influence of anthropogenic activities on the alterations of OFGs in terrestrial soils collected near airports. Moreover, the daily activities of penguins could also have caused fluctuations in some OFGs of the samples the close to the Tombolo area and Ardley Island. The findings proved the effectiveness of SR-ATR-FTIR in evaluating the potential sources of variations in the chemical constituents, especially OFGs, in Antarctic terrestrial soils.

## 1 Introduction

Antarctica is one of the most pristine places on Earth covering 14,200,000 km^2^, which is approximately 40% greater than the area under Europe. The climatic conditions in Antarctica are mainly governed by alterations in atmospheric circulation, especially by the positive pattern of the Southern Annular Mode, a prevailing atmospheric instability mechanism related to opposing pressure anomalies in southern latitudes [1]. After almost 30 million years of continental segregation, which resulted from its extraordinary weather conditions, polar vortex, and marine ice sheet, Antarctica is currently subjected to anthropogenic hazards, including toxic pollutant emissions from several human activities [2]. Although military activities have been conducted before the Antarctic Treaty, which came into effect on 23 June 1961, army personnel and instruments can be employed to some extent only for scientific research or any other peaceful applications on this continent [3]. Furthermore, approximately 80 research areas with 53 scientific stations, are located mainly in the coastal areas of the Antarctic continent [4,5]. Despite the unprecedented lockdown during the global pandemic, 74,401 tourists visited Antarctica during 2019–20, which was of 134% more than the tourist proportion in 2010–11 [6].

Under the scenario of increasing anthropogenic impacts in Antarctica, implementing a highly influential policy that includes a broad spectrum of measures is crucial to ensure extensive preservation of the Antarctic ecosystem. This is the elemental foundation of the Antarctic Treaty System. All anthropogenic activities mentioned in the Antarctic Treaty and their adverse ecological effects are regulated through the Madrid Protocol, which entered into force in 1998 [7]. Thus, understanding the spatiotemporal distributions of chemical pollutants in Antarctica is pivotal in conducting ecotoxicological risk assessments. Previous studies have extensively investigated the spatial distribution of polycyclic aromatic hydrocarbons (PAHs) in King George Island (KGI), Antarctica [[8], [9], [10]]. Although cancer and non-cancer risks from ecological exposure to PAHs based on the ‘Role of the Baseline Risk Assessment in Superfund Remedy Selection Decisions’ were found in the ‘acceptable level’, the percentage contribution of phenanthrene (Phe) was unusually high (50%) [8]. Moreover, the 3–4 ring PAHs showed a higher ecological risk than the 5–6 ring PAHs in KGI terrestrial soils, and the magnitudes of these ecological risks were categorised as low compared with those reported in other studies conducted in other locations [10]. Although the diagnostic binary ratios highlighted petrogenic emissions as the major contributor of PAHs, the receptor model indicated that electricity generators (22.84%) and light-duty gasoline (18.94%) were major contributors of PAHs in KGI [9].

To understand the behaviour of oil spills, such as the mechanisms influencing their chemical characteristics and level of alterations, chemical information-based strategies are important to prevent contamination and minimise any negative impacts on Antarctica's comparatively vulnerable ecological systems [11]. Several chemical compounds (e.g. *n*-alkanes, steranes, pentacyclic triterpenoids, and alkyl PAHs) have been quantitatively analyzed with respect to their adverse ecotoxicological effects on the KGI terrestrial soils [12]. Moreover, owing to extremely cold sub-Antarctic weather conditions, fossil-fuel-originating geochemical compounds have been found to persist over long periods [12].

Over the past few years, analytical techniques, such as ion chromatography, high-performance liquid chromatography, and gas chromatography mass spectrometry, have promoted research on the chemical characterisation of OFGs, such as water-soluble ionic species (WSIS), PAHs, and dioxins, in different environmental media [9,10,[13], [14], [15], [16], [17]]. Although these techniques exhibit high sensitivity and selectivity, they also have some disadvantages. For instance, they require a long analytical time with sophisticated optimisation and can cause destruction to target environmental samples. Hence, numerous studies have utilized synchrotron radiation-based attenuated total reflection-Fourier transform infrared (SR-ATR-FTIR) spectroscopy to characterise OFGs in several environmental components, including the epicuticular wax layer on plant leaf surface, and spore and pollen samples [[18], [19], [20], [21]]. Compared with most chromatography techniques, SR-ATR-FTIR is relatively less time-consuming, has financial benefits, requires minimal sample preparation, and is non-destructive.

SR-ATR-FTIR spectroscopy has brought about a fundamental change in the analysis of OFGs in terrestrial soils. It offers a number of innovative features and benefits over conventional approaches.

(i) **High-resolution imaging:** Due to diffraction constraints, traditional FTIR techniques can only provide limited spatial resolution. On the contrary, SR-FTIR can provide microspectroscopic information with a spatial resolution in the micrometer range by harnessing the highly collimated and strong light source from synchrotron radiation. This enables thorough chemical characterisation of sophisticated soil microstructures and OFG spatial distribution.
(ii) **Enhanced Sensitivity:** In comparison to traditional FTIR, SR-ATR-FTIR provides a much greater brightness. This enhanced sensitivity ameliorates the capability to define and quantify minute soil components by enabling the chemical characterisation of trace levels of OFGs.
(iii) **Depth Profiling:** While classical ATR-FTIR may struggle, SR-ATR-FTIR can generate precise depth profiles of terrestrial soil samples, providing insights into the vertical distribution of OFGs in the soil layers.
(iv) **Non-destructive Analysis:** The original sample integrity is preserved by SR-ATR-FTIR, unlike some terrestrial soil characterisation techniques that necessitate extensive sample preparation or destruction.
(v) **Rapid Data Acquisition:** High-throughput chemical characterisation of terrestrial soil samples is made possible by SR-ATR-FTIR's ability to capture data at a pace that is substantially faster than that of traditional FTIR due to the high-intensity synchrotron light source.
(vi) **Multiplex Advantage:** Multiple OFGs can be analyzed using SR-ATR-FTIR in a single detection by simultaneously collecting data across a broad spectral range.

In addition, defining OFGs in terrestrial soils using SR-ATR-FTIR has the potential to provide thorough, high-resolution information on terrestrial soil composition and structure. SR-ATR-FTIR is a crucial tool for expanding our insights of soil organic matter dynamics and soil health because of the technique's non-destructive nature, increased sensitivity, fast data collecting, and in situ detecting capabilities.

When applied to environmental samples, researchers can learn more about the chemical characterisation of samples in depth with the aid of SR-ATR-FTIR. Here are some applications:

(i) **Chemical Characterisation of Air Pollutants**: SR-ATR-FTIR can be applied to analyse airborne particulate matter collected from different atmospheric environments. The technique can categorise the chemical composition of particulate matter, which can include organic and inorganic compounds, providing valuable information for source apportionment [20,22].
(ii) **Soil and Sediment Characterisation:** SR-FTIR can shed light on the organic and mineralogical components of terrestrial soils, marine deposits and riverine sediments [19,23,24]. This application is also effective for characterising chemical pollutants like heavy metals, persistent organic pollutants (POPs), and microplastics within these samples.
(iii) **Water Contaminant Analysis:** Water samples can be comprehensively investigated to categorise and quantify pollutants such as microplastics, pharmaceuticals, and other toxic contaminants [25,26]. SR-ATR-FTIR can give an extensive profile of these pollutants, enabling policy makers to launch the most appropriate clean-up efforts and pollution prevention strategies.
(iv) **Microbial Community Analysis:** In environmental microbiology, SR-ATR-FTIR can be applied to rapidly identify and differentiate microorganisms, comprehend their functions in biogeochemical cycles, or predict their reactions to contamination or environmental changes [27,28].
(v) **Biogeochemical Cycling:** SR-ATR-FTIR can assist with the understanding of biogeochemical cycling processes, such as the carbon and nitrogen cycles, which are crucial for the ecosystem's health by examining the molecular features of OFGs in environmental samples [29,30].
(vi) **Biomonitoring:** SR-ATR-FTIR can be applied to examine plants, lichens, and other creatures to study how they accumulate and react to toxic chemicals, providing crucial information related to the environmental health of particular places [31].

By utilizing SR-FTIR in these applications, geochemical scientists can acquire an insight of the chemical composition of environmental samples. The resulting data can inform efforts to mitigate pollution, protect ecosystems, and better understand the environmental impact of anthropogenic activities.

Although various studies have demonstrated the promising applications of SR-ATR-FTIR for chemical characterisation of particulate OFGs in ambient air, terrestrial soils, and riverine sediments, the influence of anthropogenic activities on the spatiotemporal distribution of OFGs in the terrestrial soils of KGI have been rarely studied. To bridge this gap, this study aimed to: (*i*) chemically identify OFGs in the terrestrial soils of KGI using SR-ATR-FTIR, (*ii*) assess the impacts of anthropogenic activities on the spatial distribution of OFGs in the terrestrial soils, and (*iii*) use advanced statistical algorithms to evaluate the potential contributors of the detected OFGs.

## 2 Materials and methods

### 2.1 Site description & terrestrial soil collection

**Fig. 1:**
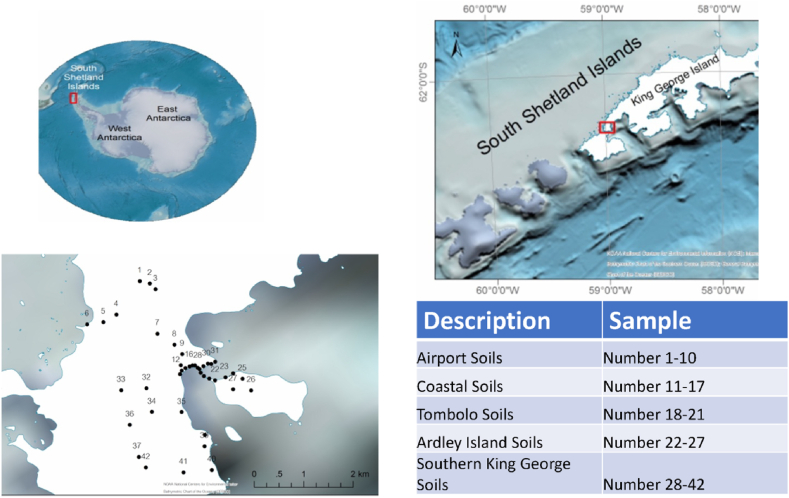
Sampling locations of the 42 terrestrial soil samples.

As part of the 34th Chinese National Antarctic Research Expedition, 42 terrestrial soil samples were collected from near the Teniente Rodolfo Marsh Martin airport (ICAO: SCRM) (*n* = 10), coastline (*n* = 7), Tombolo area (*n* = 4), Ardley Island (*n* = 6), and Southern King George (*n* = 15) (Fig. 1). SCRM airport on KGI, is considered the northernmost airport in Antarctica. It is adjacent to the village of Villa Las Estrellas and Base Presidente Eduardo Frei Montalva. On KGI, there are ten permanent Antarctic research stations: Comandante Ferraz, Arctowski, Jubany, King Sejong, Great Wall, Artigas, Bellingshausen, Eduardo Frei, Julio Escudero, and Estación Marítima Antártica. Although the atmospheric fine particle burden in Antarctica can be attributed to long-range forest fire emissions at lower southern latitudes [32,33], the adverse ecological impacts of anthropogenic activities in the continent are undeniable. For instance, a fire at the Comandante Ferraz Antarctic station on 25 February 2012 resulted in the combustion of fossil fuels that in turn generated several persistent organic pollutants, such as PAHs, hexachlorobenzene, and polychlorinated biphenyls (PCBs) [34]. A recent study confirmed the occurrence and bioaccumulation of organochlorine pesticides, PCBs, polychlorinated naphthalenes, hexabromocyclododecanes, and Dechlorane Plus in animal samples collected from KGI [35,36]. Therefore, spatially balanced soil sampling was conducted across the Fildes Peninsula, which is a 7 km long peninsula that forms the southwestern end of KGI in the South Shetland Islands of Antarctica. In this study, terrestrial soil samples (*n* = 42) were obtained from 0 to 10 cm soil depths using a hand-held core drilling device. The sampling positions of each sample are shown in Fig. 1. After sample collection, terrestrial soils were covered with dichloromethane pre-washed aluminium foil, placed in glass bottles, and stored at −20 °C [9,10].

### 2.2 SR-ATR-FTIR analysis

While both SR-ATRFTIR and Gas Chromatography-Mass Spectrometry (GC-MS) are powerful analytical instruments, there are a few reasons why SR-FTIR can be acknowledged more environmentally friendly when it comes to chemically characterizing OFGs:

(i) **Sample Preparation**: Significant sample preparation is frequently needed for GC-MS, including sample extraction, concentration, and occasionally derivatization. Organic solvents, which can be toxic to the environment and the analyst, are frequently used in this process. On the contrary, SR-ATR-FTIR employs fewer potentially hazardous solvents and requires little sample preparation.
(ii) **Non-Destructive Analysis**: Since SR-ATR-FTIR is a non-destructive technique, the sample is essentially unaltered after chemical analysis. This makes it possible to preserve the sample or do additional tests. On the other hand, because the material is ionized throughout the process, GC-MS is a destructive approach.
(iii) **Consumables**: Carrier gases are essentially required in GC-MS analysis, and additional consumables like capillary columns and septa may also be needed. These continuing requirements may have an adverse impact on the environment. The operational requirements of SR-ATR-FTIR, on the other hand, are more energy-based and don't call for a constant supply of consumables.
(iv) **Energy Consumption**: The operation of a gas chromatograph and a mass spectrometer in GC-MS can be highly energy-intensive, in spite of the fact that both analytical methods need energy to function. SR-ATR-FTIR can be considered as less energy-intensive, especially when performed with a contemporary, effective synchrotron source.
(v) **Waste Production**: Chemical waste from GC-MS is generated in the form of solvent waste and other components utilized in sample preparation (e.g. Soxhlet extraction, fractionization). Care must be taken when disposing of these organic solvents to avoid contaminating the environment. SR-ATR-FTIR, in contrast, creates little to no chemical waste.
(vi) **Versatility of Functional Group Analysis**: Although GC-MS is generally considered as excellent for measuring volatile and semi-volatile organic compounds, it can struggle with highly polar or larger molecular weight compounds without significant sample preparation. SR-ATR-FTIR, on the other hand, can directly analyse a wider variety of functional groups without the need for complex sample preparation or derivatization.

Therefore, from an environmental perspective, SR-FTIR is often a more sustainable choice for the analysis of OFGs in environmental samples.

**Fig. 2:**
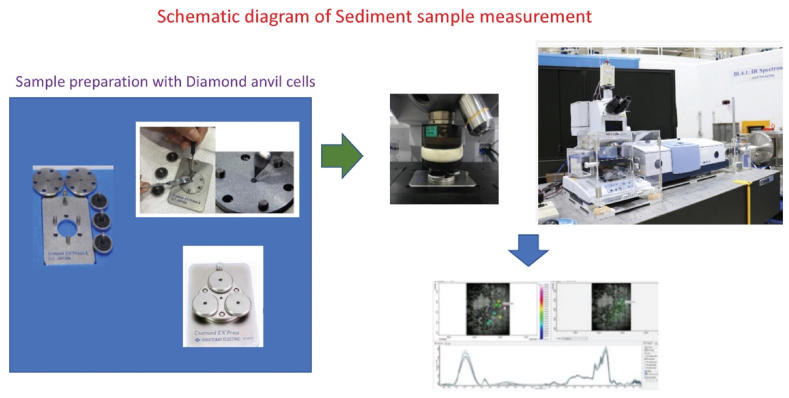
Schematic diagram of infrared micro-spectroscopy beamline (BL4.1 Infrared Spectroscopy and Imaging) using Vertex 70 FTIR spectrometer (Bruker Options, Ettlingen, Germany) coupled with infrared microscopy (Hyperion 2000, Bruker).

The collected terrestrial soil samples were prepared using a ball mill and dried overnight at 32 °C. After drying was complete, each soil sample was flattened and compressed using a diamond anvil cell (DAC), which is a high-pressure device (up to approximately 100–200 GPa) applied in geology, engineering, and material science experiments. SR-ATR-FTIR spectra were obtained by measuring each sample as a sandwich between the two diamond plates. The spectra were obtained using a Vertex 70 FTIR spectrometer (Bruker Options, Ettlingen, Germany) coupled with infrared microscopy (Hyperion 2000, Bruker) (Fig. 2). The mercury-cadmium-telluride detector was cooled with liquid nitrogen at the Beam Line 4.1 IR spectroscopy and Imaging (BL 4.1), which was specially designed to extract far-to mid-infrared light (spectral range 4000–100 cm^−1^) from the 1.2 GeV Siam Photon Source. This beamline can be further divided into three branches to ensure that the three end stations work simultaneously. The transmission mode of the sample measurements was acquired using a DAC, which was plated on a software-controlled microscope stage and placed in a specially designed box purged with dry air. The measurement was set up at an aperture of 20 × 20 μm^2^ with a spectral resolution of 4 cm^−1^, and 64 scans were co-added. The contents of WSIS and OFGs, such as aliphatic carbons (R–H), carbonyl species - hemicellulose - pectin - lectin (C=O), organo-nitrates (R–ONO_2_), aromatic nitro compounds (Arom-NO_2_), ammonium ions (NH_4_^+^), carbonate (CO_3_^2−^), nitrate ions (NO_3_^−^), sulphate species, sulphate ions, bisulphate ions (S=O), and calcium carbonate (CaCO_3_) were measured. Additionally, the relative integral areas (%) of infrared absorption bands of averaged representative spectra of terrestrial soil samples were quantified using OPUS 7.2 software (Bruker Optics Ltd, Ettlingen, Germany).

### 2.3 Quality control and quality assurance (QA/QC)

#### 2.3.1 Terrestrial soil samples

QA/QC of terrestrial soil sample analysis is crucial for ensuring accurate and reliable outcomes. Here are numerous strategies that were employed to maintain high quality control in the middle of investigation and analysis of terrestrial soil samples. Firstly, the stratified random sampling (SRS), which is a technique of sampling that includes the division of a population into smaller subgroups, was applied for collecting terrestrial soil samples in King George Island (KGI). It is also important to note that the SRS was used consistently to avoid errors due to some differences in collection techniques. Secondly, to avoid contamination or chemical alteration, terrestrial soil samples were placed in sterile containers and transported carefully and stored in a cold and dry conditions. Thirdly, all samples were labeled with the necessary information (e.g. the sampling latitude and longitude, depth, and date of sampling) to maintain the integrity of the samples until chemical analysis.

#### 2.3.2 SR-ATR-FTIR spectral

As a part of QA/QC, it is crucial to conduct performance qualification (PQ) tests to maintain the SR-ATR-FTIR spectra quality. The operator had to run PQ test regularly to ensure that the instrument is capable of quantitative chemical analysis. In this study, polystyrene was used as a standard reference material for verification of the wavenumber scale. The PQ test protocol was used to show the results of Signal to Noise Ratio (SNR) and resolution test. The SNR was measured to guarantee the performance of the SR-ATR-FTIR spectrometer. For instance, the minimum and maximum of SNR, the interferogram peak test, the wavenumber accuracy test etc. The single sample was measured for three replicates. Each replicate collected 15 spectra therefore the total spectra was 45 spectra per group. The spectra were subsequently averaged into three groups to represent the alteration of terrestrial soil samples collected from different sampling points. The number of scans for each spectrum is 64 scans number so the total sum of scans number for each replicate is 960 scans per replicate which is considerably high to get the good quality of the spectra.

### 2.4 Statistical analysis

The second derivative of SR-ATR-FTIR was employed to interpret the SR-ATR-FTIR spectra using Eq. (1). The second derivative is defined as the derivative of the first derivative. Given a function *f*, its derivative is a new function that can be described as follows:

(1) $$y=f^{\prime }\left(x\right)=\lim_{h\to 0}\left(\frac{f\left(x+h\right)-f\left(x\right)}{h}\right)$$

where *x*, *y*, *h*, and $f^{\prime }$ stand for the absorbance of the SR-ATR-FTIR spectra, f(x), its increment (i.e. $x_{1}-x_{0})$, and the first derivative, respectively. The quotient [f(x+h)−f(x)]/h indicates the limit as *h* approaches 0. Generally, the second derivative can be described by the limit definition of the derivative of the first derivative as displayed in Eq. (2):

(2) $${y^{\prime }=f}^{\prime \prime }\left(x\right)=\lim_{h\to 0}\left(\frac{f^{\prime }\left(x+h\right)-f^{\prime }\left(x\right)}{h}\right)$$

where $y^{\prime }$ and $f^{\prime \prime }$ indicate $f^{\prime \prime }\left(x\right)$ and second derivative, respectively. To assess the occurrence probability of various OFGs in the Antarctic terrestrial soils, probability distribution function (PDF), which is a mathematical function that explains the comparative likelihood for this random parameter to take on a given value, was applied. The probability of a random parameter falling within a certain region is provided by Gaussian distribution, which can be defined as described in Eq. (3):

(3) $$y=\frac{1}{\sigma \sqrt{2\pi }}\exp\left(\frac{-{\left(x-\mu \right)}^{2}}{2\sigma^{2}}\right)$$

where *y*, *σ*, *σ*^*2*^, *μ*, and *x* represent the PDF, standard deviation of SR-ATR-FTIR spectra absorbance, variance of SR-ATR-FTIR spectra absorbance, average of SR-ATR-FTIR spectra absorbance, and SR-ATR-FTIR spectra absorbance of all the KGI terrestrial soil samples, respectively.

## 3 Results and discussion

### 3.1 Characteristics of the SR-ATR-FTIR spectra

**Fig. 3:**
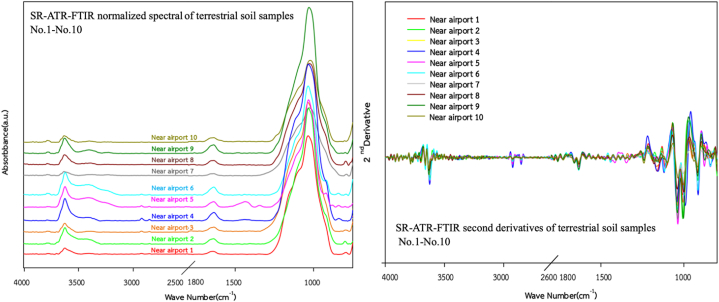
SR-ATR-FTIR normalized spectral and its second derivatives of terrestrial soil sample Nos. 1–10 (Teniente Rodolfo Marsh Martin airport) in the 4000–800 cm^−1^ frequency range.

**Fig. 4:**
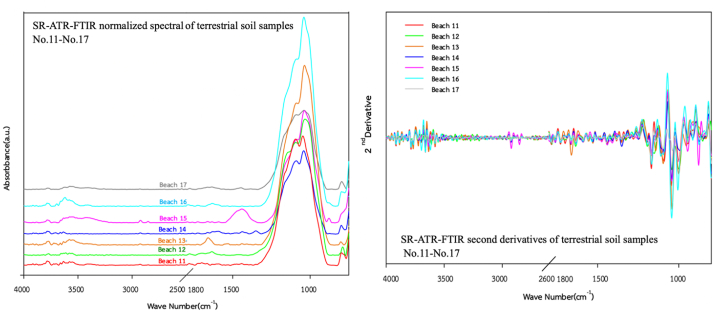
SR-ATR-FTIR normalized spectra and its second derivatives of terrestrial soil sample Nos. 11–17 (coastal soils) in the 4000–800 cm^−1^ frequency range.

**Fig. 5:**
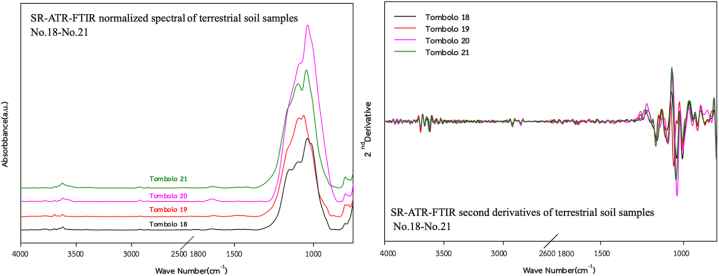
SR-ATR-FTIR normalized spectra and its second derivatives of terrestrial soil sample Nos. 18–21 (Tombolo soils) in the 4000–800 cm^−1^ frequency range.

**Fig. 6:**
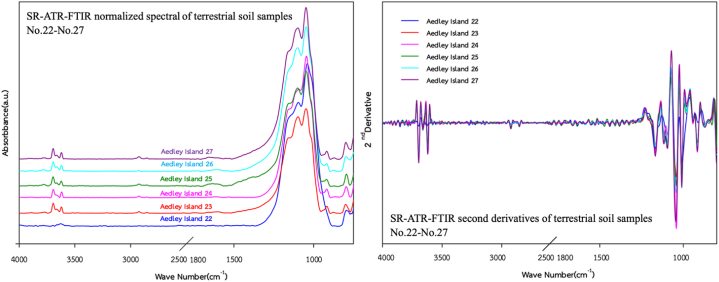
SR-ATR-FTIR normalized spectra and its second derivatives of terrestrial soil sample Nos. 22–27 (Ardley Island soils) in the 4000–800 cm^−1^ frequency range.

**Fig. 7:**
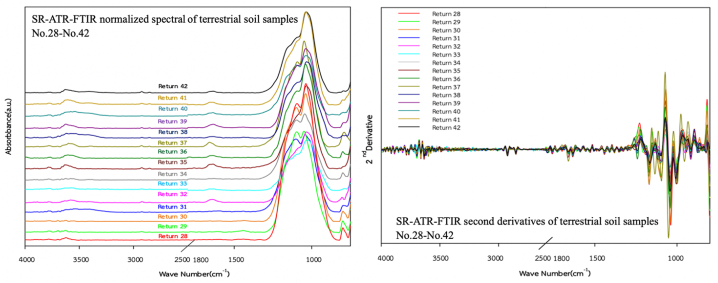
SR-ATR-FTIR normalized spectra and its second derivatives of terrestrial soil sample Nos. 28–42 (Southern KGI) in the 4000–800 cm^−1^ frequency range.

To evaluate the capability of SR-ATR-FTIR spectra as geochemical tracers to classify OFG-contaminated terrestrial soils, all samples were categorised into five zones, namely Zone-1 (Teniente Rodolfo Marsh Martin airport; *n* = 10), Zone-2 (coastline; *n* = 7), Zone-3 (Tombolo area; *n* = 4), Zone-4 (Ardley Island; *n* = 6), and Zone-5 (Southern King George; *n* = 15). The SR-ATR-FTIR spectra for all the sampling zones are illustrated in Fig. 3, Fig. 4, Fig. 5, Fig. 6, Fig. 7. Although the SR-ATR-FTIR spectra of all Antarctic terrestrial soil samples exhibited similar distribution patterns in the frequency range of 4000–800 cm^−1^, some minor differences were observed in the vibrational modes of organic components, indicating dissimilarities in the chemical compositions in each sampling zone. For instance, the SR-ATR-FTIR spectra of the Zone-1 samples were quite similar to each other, which can be characterised by: *i*) sharp and medium absorption peaks at 3700–3584 cm^−1^ interval (O–H stretching of alcohol) followed by medium N–H stretching bands at 3400–3300 cm^−1^ interval of aliphatic primary amine; *ii*) medium C–H stretching of alkane absorption peaks in the 3000–2840 cm^−1^ frequency range; and *iii*) overlap of several potential absorption peaks, namely C–O stretching of primary alcohol, S=O stretching of sulfoxide, and CO–*O*–CO stretching of anhydride in the frequency ranges of 1085–1050 cm^−1^, 1070–1030 cm^−1^, and 1050–1040 cm^−1^, respectively (Table 1 and Fig. 3). A previous study using 50 representative crudes from various Colombian oil exploitation zones and 12 samples from different Iranian oil fields highlighted a prominent feature of the absorption ATR-FTIR peak at 2920–2850 cm^−1^, which was in good agreement with the spectra of the samples collected from Zone-1 (i.e. No. 1–6) [37,38]. Moderately strong absorption ATR-FTIR peaks at 3000–2800 cm^−1^, which represented aliphatic organic matter, were also detected in oil shales collected from Australia, Brazil, Estonia, Morocco, Scotland, Sweden, and the USA [39].

**Table 1:** FTIR absorptions for representative OFGs in the terrestrial soils of KGI, Antarctica [40,41].

Wavenumber (cm^−1^)	Functional groups	Source
3750–3000	OH vibration	Hydroxyl groups in aluminosilicates and OH vibration of water and OH groups organic fraction of sediment
3000–2800	CH stretching	Aliphatic carbon – CH vibrations
1750–1700	C=O ester	Broadening and shifts of the carbonyl absorption have been observed Other types of carbonyls, e.g. ketones, aldehydes, lactones, and anhydrides, might play a role in the broadening of the carbonyl bond
1680–1600	C=C	C–C stretching of aromatics and alkene double bonds
1600–1570	COO-	COO- asymmetric stretching of metal carboxylates and carbonate minerals (calcite)
1460–1400	CH bending	Absorption peak at 1460–1418 cm^−1^ resolves into the CH bend vibration (1467 cm^−1^) as along with peaks at 1415 and 1376 cm^−1^
1200–900	Si–O	Silicate mineral

Although SR-ATR-FTIR is a promising analytical technique that can detect the spatial distribution of OFGs within polar terrestrial soils, analysing spectroscopic data is problematic because absorption peaks frequently overlap with each other. To resolve this issue, second-derivative spectroscopy, a statistical process, which separates overlapping SR-ATR-FTIR peaks, has been widely used in numerous studies for several decades [[42], [43], [44]]. Second-derivative spectroscopy enables greater classification of small and nearby SR-ATR-FTIR absorption peaks which cannot be resolved in the original spectrum. Consequently, this spectroscopic technique provides a statistical approach to enhance the selectivity of absorption peaks for specific OFGs in Antarctic terrestrial soil samples. Another advantage of this method is that constant and linear components of baseline errors are eliminated during differentiation [44], which enhances the reliability of second-derivative spectroscopy for statistical analysis. Furthermore, it eliminates undesired modifications, such as baseline shifts, increases in signal properties, and decreases undesired spectral features by broadband components [45]. In this study, the second-derivative transformation with Savitzky-Golay smoothing, focusing on the 4000–800 cm^−1^ region of the spectra, was conducted.

The second derivatives of SR-ATR-FTIR spectra of terrestrial soil sample No. 22–27 (Ardley Island soils) exhibited some remarkable differences in the frequency ranges of 1118–1098 cm^−1^, 1240–1160 cm^−1^, 1377 cm^−1^, and 3750–3000 cm^−1^ assigned to —C—O (stretching), —C—O (ester) coupled with –CH_2_— (stretching and bending), —C—H (CH_3_: bending (symmetric)), and OH vibrations, respectively (Fig. 6). According to Pietr et al. (1983) [46], who studied the mineralisation of penguin excrement in the Admiralty Bay region at KGI, ‘white’ fraction of uric acid contained the ‘red’ fraction of krill exoskeleton with a high degree of chitin (65.8%) and phosphorus (2.45%). Antarctic terrestrial soils are nutrient-poor ecosystem [47]. The main contributors of organic materials (e.g. C, N, and P) are guano, eggshells, feathers, and penguin excrement, which subsequently result in ornithogenic soils [48]. Furthermore, a recent study on the chemical characterisation of OFGs detected in *Deschampsia antarctica*, which is one of the two flowering plants commonly found in Antarctica, highlights the importance of phenols (up to 900 mg L^−1^), including hydroxybenzoic acids, hydroxycinnamic acids, and their derivatives (e.g. C_11_H_10_O_5_ (*p*-Coumaroyl glycolic acid), C13H12O8 (*p*-Coumaroyl tartaric acid)), flavonoids and their derivatives (e.g. C_15_H_8_O_5_ (Coumestrol), C_15_H_14_O_5_ (Tetrahydroxyfla van/Tetrahydroxy dihydroxychalcone)), and other compounds, such as fatty acids [49]. Overall, the prominent feature of second-derivative spectra detected in the frequency ranges of 1118–1098 cm^−1^, 1240–1160 cm^−1^, 1377 cm^−1^, and 3750–3000 cm^−1^ could be attributed to the comparatively high contents of chitin, phenols, flavonoids, and other fatty acids in the ornithogenic soils of Ardley Island.

### 3.2 Contribution of OFGs in the KGI terrestrial soils

Most SR-ATR-FTIR absorbances of samples were concentrated in the frequency range of 1200–750 cm^−1^ (Table 2) with a contribution of >75%. Comparatively high contributions of the O–H vibration group were detected in Zone-1 and Zone-2 (21.2% and 11.1%, respectively). Interestingly, the contribution of C=C observed in Zone-1 was approximately four times higher than those of Zone-2 and Zone-3. Vasiliadou et al. (2011) reported an absorption band at approximately 910 cm^−1^ assigned to the Si–O–Si bending vibration, and absorption bands at 997, 1,025, and 1115 cm^−1^ to the Si–O stretching vibrations [50]. The vibrations of water molecules sorbed onto kaolinite showed the highest peaks in the frequency ranges of 3,618, 3,650, and 3690 cm^−1^ [50]. Further, Palayangoda and Nguyen (2012) reported the IR absorption band features of illite as a strong absorbance at 980 cm^−1^, followed by comparatively medium absorbance at 794 cm^−1^ and relatively weak absorbance at 1477 and 3601 cm^−1^ [51]. Hence, the SR-ATR-FTIR absorbance of soil samples collected near the airport can be considered as a mixture of kaolinite and illite. This was in good agreement with an earlier investigation, which geochemically characterised illite and kaolinite as two major clay minerals detected in the phyllic-argillic zones of the KGI, South Shetland Islands, West Antarctica [52]. Moreover, a similar study on geochemical classification with the assistance of *X*-ray diffraction of cryosols from ice-free areas of Admiralty Bay, KGI, highlighted the role of chemical weathering in the formation of kaolinite, chlorite, and illite, which supports this interpretation [53].

**Table 2:** Contribution (%) of averaged OFGs in the KGI terrestrial soils as detected by SR-ATR-FTIR.

Sampling area	O–H vibration 3750–3000 cm^−1^	C–H stretching 3000–2800 cm^−1^	C=O 1750–1700 cm^−1^	C=C 1680–1600 cm^−1^	COO^−^1600–1570 cm^−1^	C–H bending 1460–1400 cm^−1^	Si–O 1200–750 cm^−1^
Airport	21.22	0.12	0.02	0.95	0.01	0.14	77.54
Coastal zone	11.11	0.11	0.07	0.29	0.02	0.17	88.23
Tombolo	2.52	0.18	0.03	0.24	0.01	0.05	96.97
Ardley Island	5.03	0.16	0.04	0.13	0.01	0.07	94.56
Southern King George Island	6.85	0.13	0.03	0.34	0.01	0.09	92.56

### 3.3 PDF and Pearson's correlation analysis

**Fig. 8:**
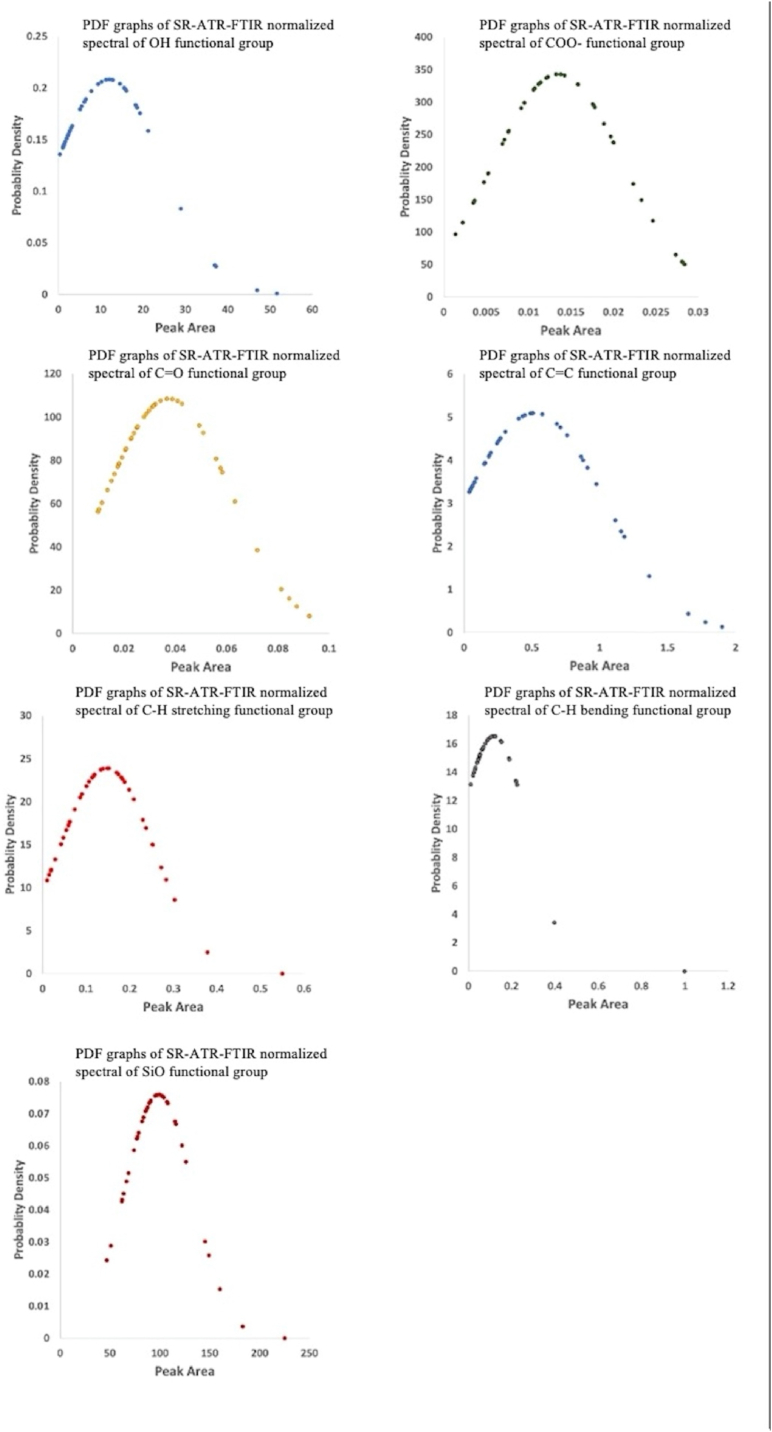
PDF graphs of SR-ATR-FTIR normalized spectral of the terrestrial soil samples.

Most PDFs of the SR-ATR-FTIR absorbances represented a positively skewed normal distribution, which can be described as a distribution where the average, median, and mode of the distribution were positive rather than negative or zero (Fig. 8). Skewness indicated a magnitude of the asymmetry of the curve plots between SR-ATR-FTIR spectra intensity and PDF of a real-valued random parameter (i.e. SR-ATR-FTIR spectra intensity). Generally, three prominent features of PDF distribution exist: positively skewed distribution, negatively skewed distribution, and undefined. Unlike the positively skewed distribution, the negatively skewed distribution tended to have more values, focusing on the right tail of the PDF curve with a comparatively longer left tail. Because the average of the PDF curve regulates the position of the distribution centre and the standard deviation controls the curve height and width, the symmetrical tall narrow bell-shape curve of the Si–O functional group represented a relatively low standard deviation with fewer outliers of this OFG. This finding was consistent with the results of previous studies that highlighted the importance of illite ((K,H_3_O)(Al,Mg,Fe)_2_(Si,Al)_4_O_10_[(OH)_2_·(H_2_O)]) and kaolinite (Al_2_O_3_·2SiO_2_·2H_2_O) as the two main clay minerals homogeneously distributed in the KGI terrestrial soils [52,53]. Contrastingly, the positive skewness of the normal distribution of OH, C=O, C=C, and C–H stretching and bending indicated the impact of positive outliners in the dataset. Further, because the height is controlled by the scaling factor and the width is governed by the factor in the power of the exponential (Eq. (3)), the comparatively broad long right tail of the PDF curve most likely represented the role of anthropogenic activities in some unusually high contents of OFGs, including hydroxyl, carbonyl, alkene, and alkyl groups. These findings indicated highly deviated OFG distributions in KGI terrestrial soils as a consequence of relatively complicated chemical mixtures plausibly derived from both local anthropogenic activities and long-range atmospheric transport.

**Fig. 9:**
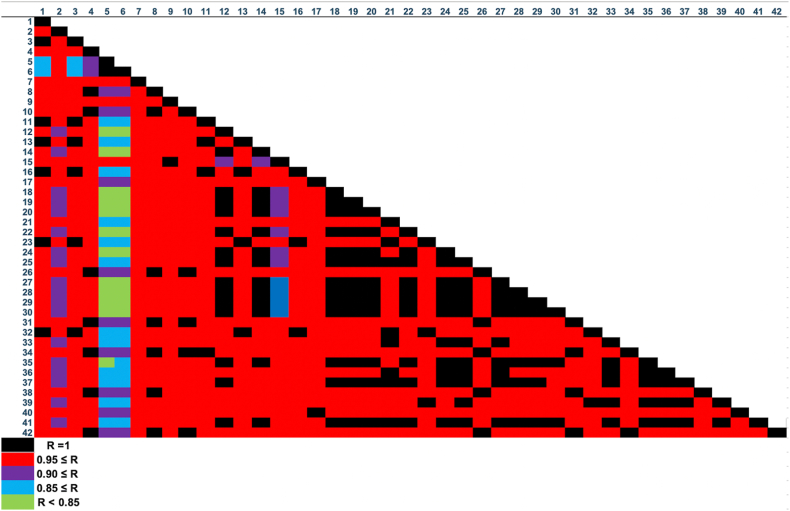
Pearson's correlation analysis of SR-ATR-FTIR normalized spectral of the terrestrial soil samples.

Correlation analysis revealed interesting relations of each OFG in KGI terrestrial soils (Fig. 9). Sample Nos. 18–30 showed extremely strong positive correlations (0.90 < r < 1.00), indicating that Tombolo and Ardley Island soils shared similar chemical distribution patterns. These findings were consistent with the fact that Penguin colonies play a crucial role as the main biota in maritime Antarctic terrestrial ecosystems [54,55]. Sun et al. (2000) reported the enrichment of nine chemical species (e.g. F, P, and Cu) in the sediments near penguin rookeries owing to the deposition of large amounts of penguin excreta along with numerous physical disturbances, such as trampling and uprooting of vegetation [56]. Hence, the strong positive correlation coefficients of the terrestrial soil samples collected from Tombolo and Ardley Island could be attributed to the daily activities of penguins. Contrastingly, comparatively weaker positive correlation (r < 0.85) was detected in sample Nos. 5 and 6 (Fig. 9), which could be attributed to some dissimilarities in the chemical matrices of these two samples compared to that of the samples. Since these two terrestrial soil samples were collected from near the airport, anthropogenic activities near the airport could be responsible for the weak correlation. Additionally, further advanced statistical analysis of these OFGs will be discussed through the hierarchical cluster analysis (HCA) and principal component analysis (PCA) results.

### 3.4 Hierarchical cluster correlation analysis

**Fig. 10:**
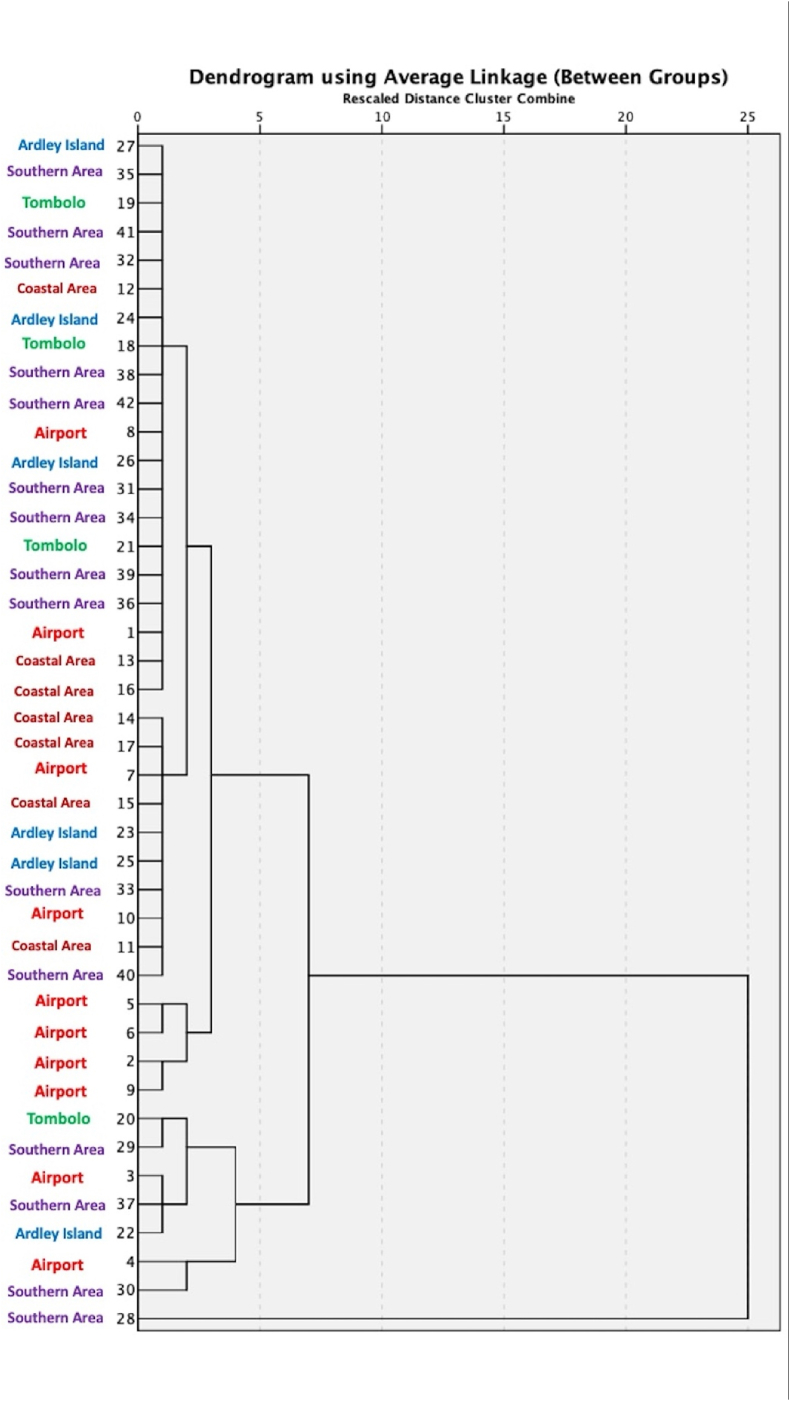
Hierarchical cluster analysis of the SR-ATR-FTIR normalized spectral of the terrestrial soil samples.

Although HCA has been widely applied in image processing, data analysis, and pattern recognition for several decades, this advanced statistical technique can assist geochemical scientists in identifying subgroups and/or chemical profiles of individual environmental samples within a larger population that share similar patterns of parameters [9,14,57]. To obtain better insights into the origins of OFGs in the Antarctic terrestrial soils, HCA was applied for 42 variables (i.e. database of 42 individual KGI soil samples) and seven parameters of the SR-ATR-FTIR absorbance spectrum (i.e. O–H vibration (3750–3000 cm^−1^), C–H stretching (3000–2800 cm^−1^), C=O (1750–1700 cm^−1^), C=C (1680–1600 cm^−1^), COO^−^ (1600–1570 cm^−1^), C–H bending (1460–1400 cm^−1^), and Si–O (1200–750 cm^−1^)). The HCA results of the 42 KGI soil samples confirmed the presence of the following two main groups as shown in the dendrogram in Fig. 10: the first cluster (*n* = 34) consists of terrestrial soil samples collected at the Southern area (*n* = 11) (i.e. Nos. 31–36, and Nos. 38–42), Ardley Island (*n* = 5) (i.e. Nos. 23–27), Tombolo (*n* = 3) (i.e. Nos. 18–21), airport (*n* = 8) (i.e. Nos. 1–2 and No. 5–10), and coastal area (*n* = 7) (i.e. Nos. 11–17). The second cluster (*n* = 8) included the remaining KGI soil samples (Nos. 3–4, 20, 22, 28–30, and 37). The first cluster classified terrestrial soils collected near the airport, with the exception of only two samples (3 and 4). Interestingly, 83% and 75% of the Ardley Island and Tombolo soil samples were present in the first main cluster. Contrastingly, the first main cluster contained only 73% of the Southern area soil samples, indicating that comparatively more complicated chemical matrices exist in the southern part of KGI, thus, limiting the classification ability of this statistical technique.

### 3.5 PCA

The principal component patterns for varimax-rotated constituents of all SR-ATR-FTIR spectra were constructed with five principal components (PCs), which explained 90.7% of the total variance (Table 3). Strong positive correlation coefficients of the hydroxyl group (0.903) and alkene (0.904) were observed in PC1, with 31.2% variance. Wang et al. (2021) reported aliphatic compounds and hydroxyl groups as the main chemical structural units of Indonesian oil-sand bitumen samples [58]. De Rivas et al. (2017) indicated that the FTIR spectra of oil samples used in private aircraft also demonstrated a large absorption band (1680–1600 cm^−1^), indicating alkene as the dominant chemical species [59]. A strong positive correlation of C–H stretching was found at PC4, with 11.5% variance. Numerous studies have reported significant peaks in the spectral ranges of 3000–2800 cm^−1^ (i.e. C–H stretching functional group) detected in various types of oil samples. Thus, PC4 can confirm oil spill pollution (e.g. crude oil, diesel fuels, aircraft fuels, kerosene, hydraulic oils, and lubricating oils) in KGI terrestrial soils [60,61]. Therefore, both PC1 and PC4 can be considered as the sum of oil spill contamination, with a contribution of 42.7%. A moderately strong positive correlation coefficient (0.79) of the carbonyl group, which is a constituent of acyl halides (RCOX), quinones (C_6_H_4_O_2_), anhydrides (−COOOC−), esters (R–COO–R′), amides (CO–NH), and carboxylic acids (R–COOH), was detected in PC2, with 24.5% variance. This can be explained by Antarctic microbial communities and several organic debris, including microbiaeratin (i.e. a new natural indole alkaloid from a microbispora aerata strain), cyclic heptapetides (C_44_H_57_N_7_O_8_) produced by the Antarctic fungus *Cadophora malorum*, and heteropolymer humic substances frequently found in maritime Antarctic tundra, all of which are commonly found in the southernmost continent [[62], [63], [64]].

**Table 3:** Varimax rotated component matrix of OFGs in the KGI terrestrial soils as detected by SR-ATR-FTIR.

	Principal Component (PC)				
	PC1	PC2	PC3	PC4	PC5
O–H vibration 3750–3000 cm^−1^	**0.903**	0.068	0.305	−0.043	−0.071
C–H stretching 3000–2800 cm^−1^	0.117	−0.073	0.139	**0.972**	0.060
C=O 1750–1700 cm^−1^	−0.175	**0.788**	0.161	−0.144	−0.178
C=C 1680–1600 cm^−1^	**0.904**	−0.184	−0.008	0.220	0.069
COO^−^1600–1570 cm^−1^	0.048	0.908	−0.091	0.027	−0.051
C–H bending 1460–1400 cm^−1^	0.201	0.032	**0.953**	0.146	−0.032
Si–O 1200–750 cm^−1^	−0.004	−0.167	−0.031	0.058	**0.982**
Variance (%)	31.20	24.45	13.44	11.54	10.04
Estimated sources					

## 4 Conclusions

Antarctica is one of the most pristine ecosystems on Earth because of relatively low impacts of human civilisation. However, its well-preserved natural environment has been significantly damaged by both natural causes, such as atmospheric deposition of chemical pollutants, and local anthropogenic activities, including scientific operations, maritime shipping, unintentional oil spills, and accidental explosion of fossil fuels. Although SR-ATR-FTIR has a comprehensive scope for non-destructive chemical analysis in scientific research, few studies have reported the spatial distribution of OFGs in Antarctic terrestrial soils. Several multivariate statistical techniques have highlighted anthropogenic activities as the main contributors (approximately 43%) of some OFGs. Moreover, some local organic debris, such as penguin excrement, coupled with secondary metabolites produced by bacteria, fungi, and plants, are responsible for almost 25% of OFGs present in the KGI terrestrial soils. In conclusion, SR-ATR-FTIR proved to be effective for the spectroscopic evaluation of several potential sources of variations in chemical constituents, especially OFGs, in Antarctic terrestrial soils.

## Funding

This work was supported by the National Institute of Development Administration-Research Centre (NIDA-RC).

## Author contribution statement

Siwatt Pongpiachan: Conceived and designed the experiments; Analyzed and interpreted the data; Wrote the paper.

Kanjana Thumanu; Muhammad Zaffar Hashmi: Analyzed and interpreted the data; Contributed reagents, materials, analysis tools or data; Wrote the paper.

Chulalak Chantharakhon: Contributed reagents, materials, analysis tools or data; Wrote the paper.

Chunmanus Phoomalee; Saran Poshyachinda: Performed the experiments; Analyzed and interpreted the data; Wrote the paper.

Teetat Charoenkalunyuta; Kittiphop Promdee: Conceived and designed the experiments; Wrote the paper.

## Data availability statement

Data will be made available on request.

## Declaration of competing interest

The authors declare that they have no known competing financial interests or personal relationships that could have appeared to influence the work reported in this paper.
